# Bartholin Gland Cyst Mimicking Rectocele: Common Pathology at an Uncommon Location

**DOI:** 10.7759/cureus.45607

**Published:** 2023-09-20

**Authors:** Satish Choudhury, Arun K Dora, Gargi Gautam, K Pushpalatha

**Affiliations:** 1 Department of Obstetrics and Gynaecology, All India Institute of Medical Sciences, Bhopal, Bhopal, IND

**Keywords:** cyst excision, mullerian cyst, rectocele, posterior vaginal wall cyst, bartholin gland cyst

## Abstract

Vaginal cysts are often encountered in gynaecological outpatient settings. These are usually asymptomatic in their initial course but become symptomatic when their size increases or they get infected. While evaluating such cases, clinical examination plays a vital role in ruling out their differential diagnoses. Imaging studies can complement clinical findings. However, in some instances, the nature of vaginal cysts may not be determined preoperatively until histopathology examination reveals it. We report here a rare case of a posterior vaginal wall cyst that presented as a mass protruding through the vagina. The clinical dilemma was the characterization of the cyst, owing to its huge size and rare location. The cyst was managed surgically by excision, and to our surprise, histopathological examination revealed it as a Bartholin gland cyst in the posterior vaginal wall, rare in its location.

## Introduction

Cysts of vaginal origin are rare and are usually incidentally diagnosed during routine gynaecological examinations. The clinical presentation depends on the size and depth of the cyst. Larger cysts are often symptomatic and cause dyspareunia, vulvar pain, pressure symptoms, or an abscess. Various categories of vaginal cysts are classified based on the histology depending on the lining epithelium of the cyst wall: epidermal inclusion cyst, Mullerian cyst, endometroid cyst, Bartholin gland cyst or abscess, Gartner’s duct cyst, and unclassified types [1]. Bartholin gland cysts constitute 27.5% of all vaginal cysts, and 2% of women develop a Bartholin gland cyst or gland abscess in their lifetime [2]. Bartholin glands are located on postero-lateral vaginal wall (four and eight o’clock position) and drain through ducts 2.0 cm to 2.5 cm long. These ducts, when obstructed, can lead to the formation of cysts, most commonly on the postero-lateral parts of the labia majora [3,4]. Contrary to the usual presentation, the Bartholin cyst in our case presented as a posterior vaginal wall cyst, which has not been reported earlier in the literature.

## Case presentation

A 43-year-old para 2 living 2 presented to the outpatient department with complaints of mass coming out of the vagina for the last two months. The size of the mass increased insidiously. There were no associated bowel or bladder complaints. There was no history of an increase in the size of the mass after straining.

**Figure 1:**
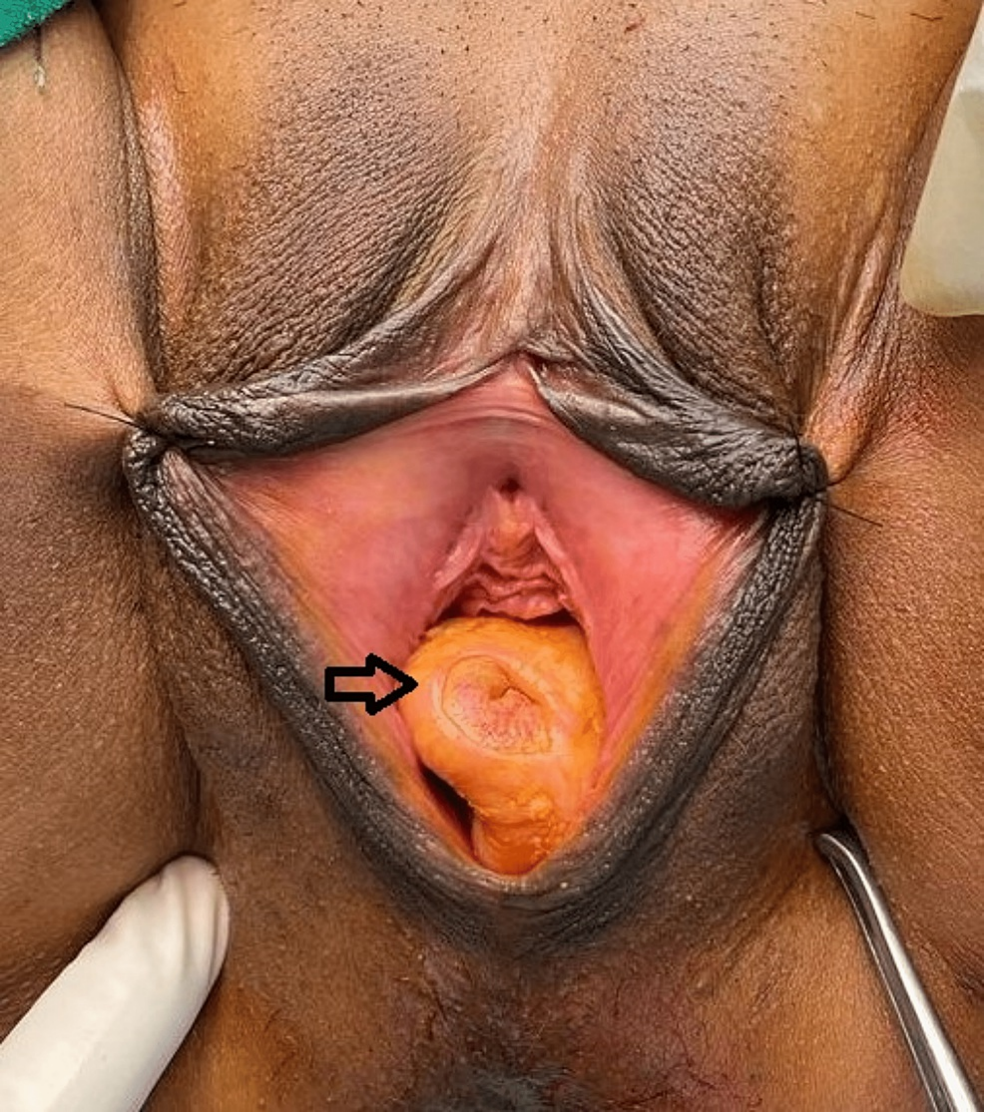
Image showing a vaginal cyst confined to the posterior vaginal wall (arrow mark)

The patient had undergone a right partial nephrectomy two years ago. Her general physical and systemic examination was unremarkable. On local examination, a cystic mass of size 6x6 cm was seen occupying the vagina and protruding through the introitus (Figure 1). Speculum examination revealed the cystic mass likely originating completely from the posterior vaginal wall. There was no associated cough impulse. On a bimanual pelvic examination, the uterus was normal in size and anteverted, and no adnexal mass could be palpated. On rectal examination, the cyst wall was found to be anterior and free from the anterior rectal wall.

**Figure 2:**
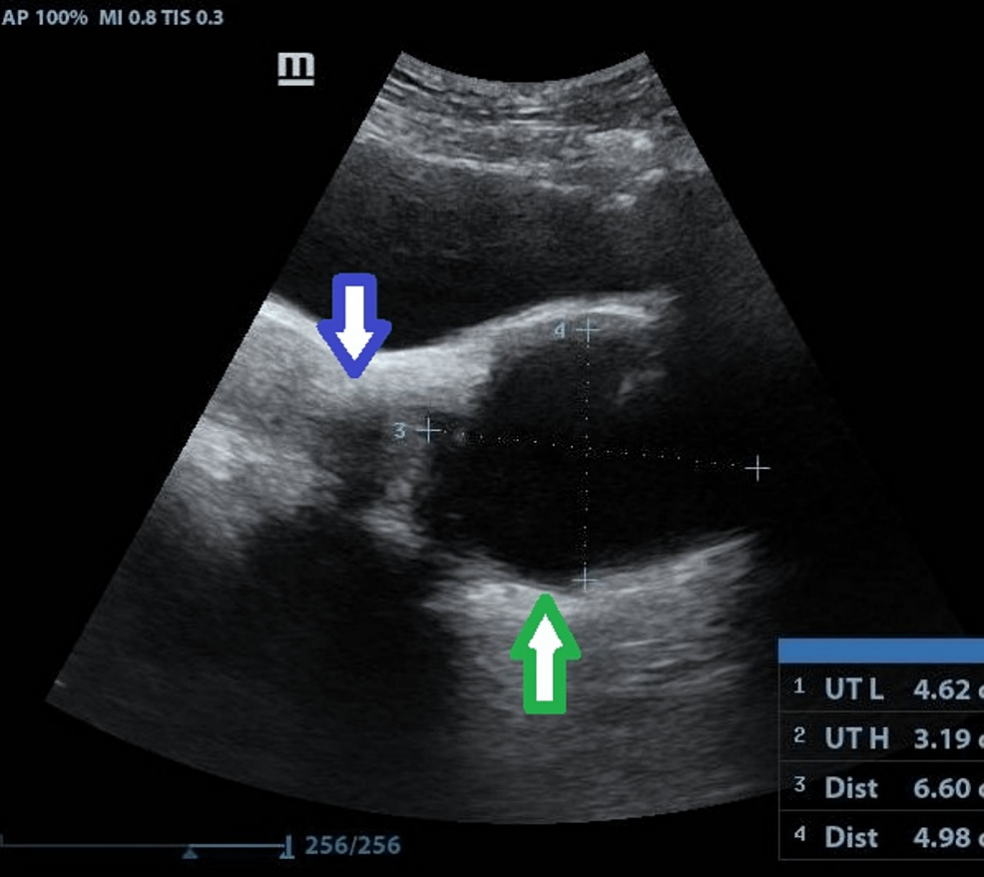
Ultrasonography shows an anechoic cyst (green arrow mark) of size 6.6 x 4.9 cm distal to the cervix (blue arrow mark) in the vaginal cavity

**Figure 3:**
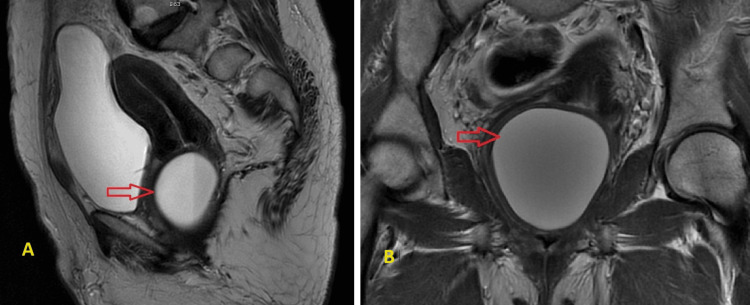
A: MRI midline sagittal view showing a well-defined thin-walled cystic lesion (red arrow mark) of size 6.6 x 5 x 5.5 cm along the posterior aspect of the mid and lower third of the vaginal wall. B: MRI coronal view showing the cyst (red arrow) compressing the vaginal canal anteriorly and abutting the rectum posteriorly.

Ultrasonography showed a normal uterus with an endometrial thickness of 6 mm. An anechoic lesion of size 6.6 x 4.9 cm was visualised distal to the cervix in the vaginal cavity; however, the origin of the lesion could not be seen (Figure 2). Magnetic resonance imaging (MRI) revealed a well-defined, thin-walled cystic lesion of size 6.6 x 5 x 5.5 cm along the posterior aspect of the mid and lower third of the vaginal wall. Anteriorly, the lesion was compressing the vaginal canal, and posteriorly, the lesion was abutting the rectum (Figure 3). Routine blood investigations were within normal limits.

A differential diagnosis of rectocele was ruled out based on the clinical examination. Enterocele was ruled out as the cyst was in the mid and lower third of the vagina and there was no associated cough impulse. The location of the cyst ruled out the Bartholin cyst, which is typically located in the postero-lateral vaginal wall, and Gartner’s cyst, which is located in the anterior or antero-lateral aspect of the vaginal wall. Inclusion cysts of the vagina are usually situated on the posterior surface, but these are small in size, and there was no history of any perineal surgery. Endometriotic cysts could be ruled out due to the absence of pain and the presence of clear cystic content based on imaging. As the cystic lesion was completely located on the posterior vaginal wall, rectal neoplasm mimicking this condition could be a possibility until clinical examination and imaging ruled it out.

**Figure 4:**
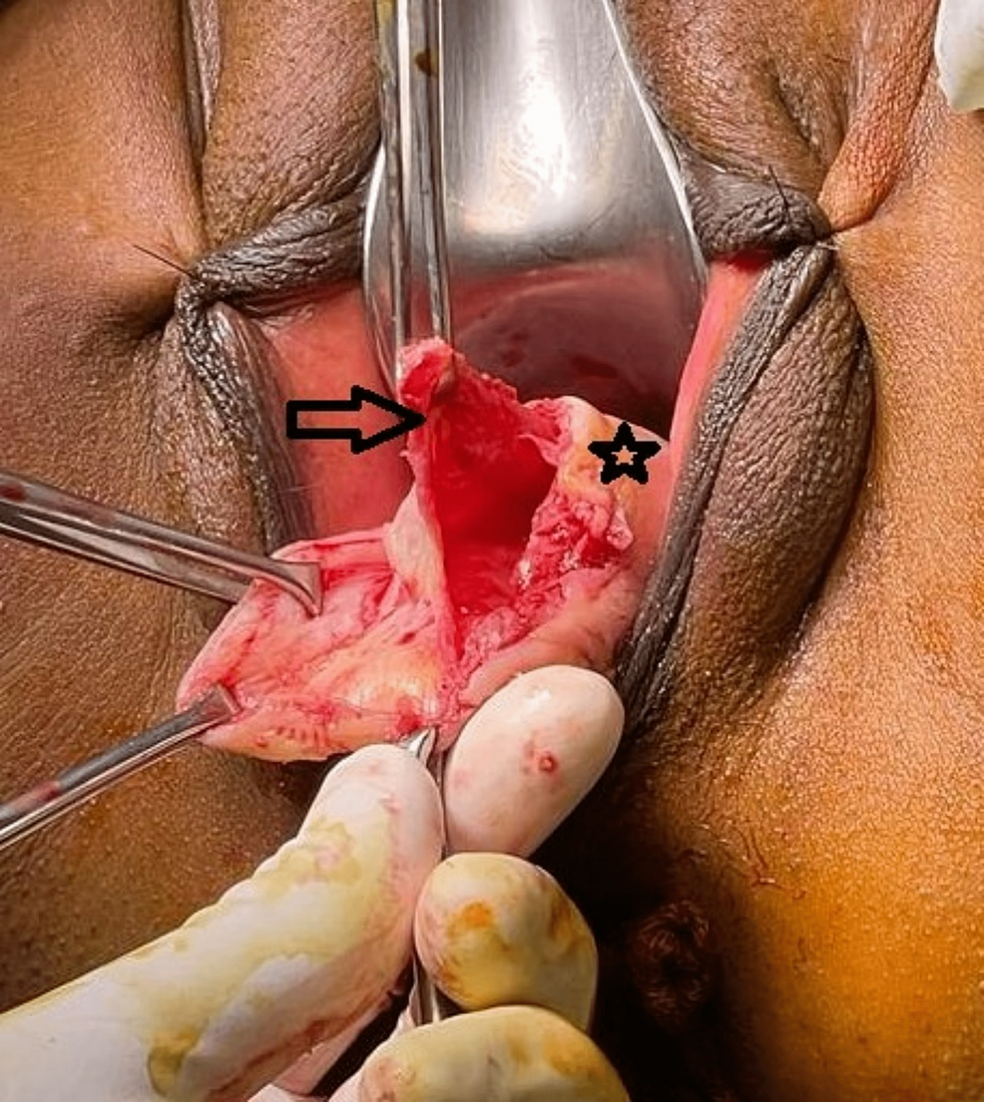
Intraoperative image showing dissection being performed to separate the cyst wall (arrow mark) from the posterior vaginal wall (asterisk mark)

**Figure 5:**
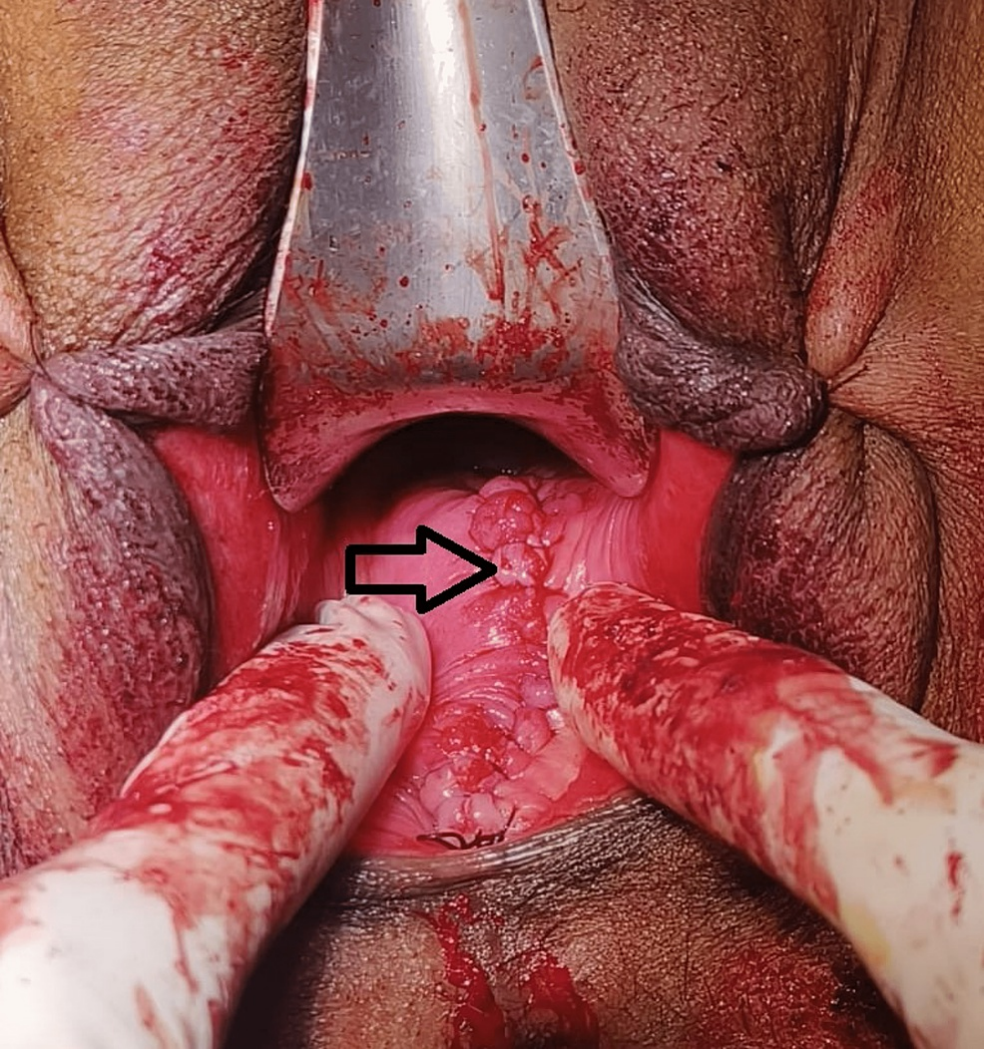
Intraoperative image showing posterior vaginal mucosa closed with absorbable sutures post-cystic excision (arrow mark)

The patient underwent surgical excision of the cyst under spinal anaesthesia. Diluted vasopressin was injected into the tissue plane between the cyst wall and posterior vaginal wall to minimise haemorrhage and hydro dissection. A vertical incision was made on the cyst, and blunt and sharp dissections were performed (Figure 4). The cyst was excised completely. Excess vaginal tissue was excised, and the posterior vaginal mucosa was closed with absorbable sutures (Figure 5). The patient was discharged in good health on the third postoperative day. The gross specimen of the cyst was grey-white in colour, and histopathology examination revealed a cyst wall lined by a low cuboidal epithelium consistent with the Bartholin cyst.

The patient was followed up for one month after surgery and had a full recovery without any complications.

## Discussion

Vaginal cysts are often diagnosed based on history and clinical examination. While performing local examinations, clinicians usually differentiate the nature and type of vaginal cyst based on the location and associated signs. Gartner cysts are usually located in the anterior or antero-lateral aspect of the vaginal mucosa. Likewise, the Bartholin gland cyst is typically located in the postero-lateral aspect of the labia majora. The Mullerian cysts arise at the level of the cervix and extend anteriorly. Various other differential diagnoses of vaginal cysts, like endometriotic cysts, inclusion cysts, rectocele, and cystocele, can also be diagnosed from history and clinical examination. In inconclusive cases, imaging modalities like ultrasonography and magnetic resonance imaging can help in delineating the origin, involvement of surrounding organs, and nature of the cyst.

Bartholin gland cysts are one of the most common incidentally diagnosed vaginal cysts among patients visiting a gynecological outpatient department. However, it sometimes presents with dyspareunia, vulvar pain, pressure symptoms, or an abscess. Bartholin gland cysts and abscesses are more likely in sexually active women due to obstruction in ducts by friction during coitus [3]. Bartholin gland cysts are typically located in the postero-lateral vaginal wall owing to their anatomical location [3]. It can extend anteriorly if it grows larger in size. Contrary to it, in our case, the vaginal cyst was completely originating from the posterior vaginal wall, which made the clinical diagnosis of the Bartholin gland cyst unlikely during its evaluation until the histopathology revealed it. A Bartholin gland cyst located on the anterior vaginal wall has been reported in the literature [5]. However, probably Bartholin gland cysts entirely located on the posterior vaginal wall have not been reported in medical literature.

The closest differential diagnosis in our case was rectocele, but it could be easily ruled out as it was not associated with utero-vaginal prolapse and the incidence of isolated rectocele is very low. On further examination, the cyst was found to be separate from the rectal wall. Imaging studies confirmed the location to be confined to the posterior vaginal wall without the involvement of surrounding structures or the anechoic content of the cyst. Imaging is often helpful in cases where malignancy is suspected, i.e., age greater than 40 years; firm, fixed, or irregular swelling. In these conditions, biopsy with or without excision is recommended [4].

The posterior location of the vaginal cyst, which mimics rectocele, clinical uncertainty about the origin of the cyst, and histopathology examination revealing it as a Bartholin gland cyst make our case an interesting and rare one.

## Conclusions

Bartholin gland cysts can occur on the posterior vaginal wall, contrary to their usual location on postero-lateral part of the labia majora. These cysts, when large enough, can present as rectocele protruding through the introitus. However, a thorough clinical examination is the key to eliminating the clinical ambiguity. Cyst excision is the standard treatment in these cases. The nature of the cyst can be confirmed by histopathology examination of the surgical specimen.
